# Frequency of Her2/*neu* expression in colorectal adenocarcinoma: a study from developing South Asian Country

**DOI:** 10.1186/s12885-016-2912-y

**Published:** 2016-11-07

**Authors:** Asma Shabbir, Talat Mirza, Abdullah Bin Khalid, Muhammad Asif Qureshi, Sadaf Ahmed Asim

**Affiliations:** 1 Sindh Medical College, Jinnah Sindh Medical University, Karachi, Pakistan; 2 Dow International Medical College, Dow University of Health Sciences, Karachi, Pakistan; 3 Dow University of Health Sciences, Karachi, Pakistan

**Keywords:** Her-2/*neu*, Colorectal adenocarcinoma, Immunohistochemistry, Trastuzumab

## Abstract

**Background:**

Human Epidermal Growth Factor (Her-2*/neu*) has strong therapeutic implications in certain cancers like breast and gastric cancer. Literature on its frequency in colorectal cancer is scarce. In this study, we have investigated the frequency of Her-2/*neu* expression in colorectal adenocarcinomas and its association with various clinicopathological variables.

**Methods:**

A total of 95 patients who underwent colonoscopic biopsy or colectomy were studied after Institutional Ethical Approval. Hematoxylin & eosin (H&E) staining was performed on all the tissue sections. Expression of Her-2/*neu* was investigated by immunohistochemistry using α-Her-2 antibody. In order to quantify Her-2/*neu* expression, three criterias were applied that includes the pattern of staining, intensity of staining and percentage of tumor cells stained. Furthermore, its association was seen with various clinicopathological variables including age, gender, histopathological type, grade and stage of the tumor. Data was entered and analyzed using SPSS version 21. A *p*-value of < 0.05 was considered as significant.

**Results:**

From the total of 95 cases, 75 (78.9 %) cases showed Her-2/*neu* expression. Pattern of Her-2/*neu* staining was significantly associated with the grade of colorectal cancer depicting cytoplasmic Her-2/*neu* expression higher in low grade (50 %) while membranous Her-2/*neu* expression more in high grade colorectal cancer (45 %) (*P*-value = 0.030). Pattern of Her-2/*neu s*taining was also significantly associated with the type of colorectal cancer representing membranous Her-2/*neu* expression to be more common in mucinous type (38.5 %) while cytoplasmic Her-2/*neu* expression to be more frequent in non mucinous type (42.7 %) of colorectal cancer (*p*-value = 0.024).

We observed a significant association between percentage of cells stained & tumor type, with score 3+ maximum in non mucinous type of colorectal cancer (*p*-value = 0.006).

**Conclusion:**

Her2/*neu* is considerably expressed in colorectal adenocarcinoma in Pakistani population. Our findings indicate a significant strong association of cytoplasmic Her-2/*neu* expression with low grades and membranous Her-2/*neu* expression with high grades of colorectal cancer. These findings add to the body of information & may help in conducting clinical trials in future to explore its therapeutic significance as well.

## Background

Gastrointestinal malignancies are amongst the major oncological problems worldwide. Colorectal cancer is the 3rd most common cancer across the globe [1]. A rise in incidence and mortality rates of colorectal cancers are illustrated in Asia [2]. In Pakistan, a low but rising incidence of colorectal cancer at young age with advanced stages is being consistently observed in the recent past [3]. Rising incidence and high mortality of colorectal cancer demands for a need of newer therapeutic targets to improve the survival of the patients.

Human Epidermal Growth Factor (Her-2*/neu*) is a proto-oncogene located on chromosome 17q21 that encodes ErbB-2 [4]. Over-expression of Her-2*/neu* has been notably associated with increased cellular survival, increased proliferation and decreased apoptotic potential of cells leading to malignant transformation and maintenance of the associated malignancy [5]. Presence of Her-2/*neu* is strongly implicated in certain cancers including breast & stomach. Trastuzumab, a monoclonal antibody against Her-2/*neu*, has shown a good prognosis in Her-2*/neu* positive breast cancer patients [6]. Recently, α-Her-2 targeted therapy has also been approved for metastatic gastric adenocarcinomas [7, 8].

A very scarce data is available particularly from our region to indicate expression of Her-2*/neu* in patients with colorectal adenocarcinomas. Overexpression of Her-2/*neu* in colorectal cancer shows a wide range of variability between 0-84 % in different studies [9].

Her-2*/neu* expresses itself in either membranous as well as cytoplasmic forms with different clinical implications in different cancers. For instance, cytoplasmic expression of Her-2*/neu* in breast cancer does occur but it has been regarded as irrelevant since the monoclonal antibodies approved for its treatment, targets only membranous forms [9]. In contrast, several studies reported that Her-2*/neu* expression to be membranous as well as cytoplasmic in colorectal adenocarcinoma [10, 11]. Moreover, literature supports that cytoplasmic Her-2/*neu* expression in colorectal carcinoma could be associated with survival prognosis [9]. The definitive cause of cytoplasmic expression of Her-2/*neu* still remains unclear but upregulation of promoter binding proteins leading to an increase in Her-2*/neu* production provides some evidence for the identity of cytoplasmic expression of Her-2/*neu* in colorectal cancer [9]. Use of α-Her-2 therapy in the treatment of colorectal cancer patients has been less extensively investigated. A clinical trial conducted showed a low positivity (8 %) of Her-2/*neu* expression but these patients responded to Trastuzumab therapy [12].

However, and alarmingly, Her-2/*neu* testing in colorectal adenocarcinoma has not gained its popularity in most parts of the world including South Asia region. Moreover, a very few studies have been published from this region regarding Her-2*/neu* expression in colorectal adenocarcinoma [13–15]. Therefore, in this study we attempted to know the expression of Her-2/*neu* in colorectal tumors in our population which might prove in future a useful prognostic factor and worthy therapeutic target for colorectal carcinoma.

## Methods

### Recruitment of patients and ethical approval

The study was conducted at the Dow Diagnostic Research and Reference Laboratory (DDRRL), Dow University of Health Sciences (DUHS), Karachi after ethical approval of Institutional Review Board during August 2014 to February 2016. A total of 95 patients with colorectal carcinoma who underwent colorectal cancer biopsy at National Institute of Liver and Gastrointestinal Diseases (NILGID) and colectomy at surgical ward of DUHS were recruited in the study after their informed consent. Clinico-pathological parameters of the patients were then recorded.

### Processing of tissues and microscopy

Subsequent to biopsy/colectomy, specimens were transferred to the Histology section in 10 % neutral buffer formalin. Samples were examined for gross features and paraffin blocks were prepared for subsequent staining and microscopy. Tissue sections, each measuring 3 to 4 μm in thickness were cut from the paraffin blocks and processed for H&E staining. Based on microscopic examination of the H&E stained slides, clinico-pathological parameters were recorded including tumor type, tumor grade amongst others. Histopathological grading of tumors was performed according to the World Health Organization (WHO) criteria as grade I (well differentiated), grade II (moderately differentiated) and grade III (poorly differentiated). Pathological staging of colectomy cases were recorded as per the 7th edition of the American Joint Committee on Cancer Staging (stage I to stage IV) [16].

### Immunohistochemistry and scoring of Her-2/*neu* over-expression

In order to investigate expression of Her-2/*neu* in colorectal adenocarcinoma, conventional immunhohistochemistry protocol was subjected on all cases. Sections were deparaffinized, dehydrated and antigen retrieval was performed. Further, the sections were incubated with the mouse primary monoclonal antibody against Her-2/*neu* (clone CB-11, dilution 1:65, Cellmarque) for 1 h in the moisturisation chamber. Secondary antibody (Hi-Def detection system) (Cellmarque) containing solution A as amplifier and solution B as polymer was used. For all read-outs, staining was controlled by using known Her-2/*neu* over-expressing breast carcinoma tissues.

Her-2/*neu* stained slides were independently evaluated by two experienced pathologists. In order to quantify/score Her-2*/neu* expression, three criterias were followed [17]:

1. Pattern of Her-2*/neu* expression: Membranous/cytoplasmic/membranous + cytoplasmic
2. Intensity of staining: Weak/moderate/strong
3. Percentage of cells stained:
  10-40 % cells stained as score 1+
  40-70 % cells stained as score 2+
  >70 % cells stained as score 3+

### Statistical analysis

Data was recorded for different variables including age and gender of the patients, histopathological type, grade, stage of the tumor and Her-2/*neu* expression and analysed using SPSS version 21. In order to investigate association of Her-2/*neu* expression in colorectal adenocarcinomas with other categorical variables, Chi-square or Fisher’s exact test were applied. A *p*-value < 0.05 was considered as significant.

## Results

### Clinico-pathological parameters of colorectal cancer patients

The study included a total of 95 cases of colorectal adenocarcinoma. Mean age of the patients recruited in this study was 46 years (range22-85 years). Of all the patients studied, a total of 51 (53.7 %) were males while 44 (46.3 %) were females. A total of 74 (77.9 %) were biopsy specimens while 21 (22.1 %) were colectomy specimens. Overall, a total of 14 (14.7 %) patients had grade I, 59 (62.1 %) had grade II and 22 (23.2 %) had grade III tumors. Histologically, 13 (13.7 %) tumors were mucinous and 82 (86.3 %) were non mucinous variants. Pathological staging could only be performed on colectomy samples (*n* = 21). A total of 9 (42.9 %) cases were of stage IIA, 1 (4.8 %) case of stage IIB, 2 (9.5 %) cases of stage IIIA, 3 (14.3 %) cases of stage IIIB & 6 (28.6 %) cases of stage IIIC.

### Her-2/*neu* Immunoreactivity in Colorectal Cancer

Of the total 95 colorectal cancer tissues, 75 (78.9 %) showed Her-2*/neu* staining. Out of the total 75 positive cases, 20 (26.6 %) showed membranous Her-2*/neu* expression, 36 (48 %) cases showed cytoplasmic expression and 19 (25.3 %) cases showed membranous + cytoplasmic expression.

Of the total 75 positive cases, 19 (25.3 %) cases showed weak intensity, 35 (46.6 %) showed moderate intensity & 21 (28 %) showed strong intensity of Her-2/*neu* staining.

Of the total 75 positive cases, 23 (30.6 %) were scored as 1+, 18 (24 %) cases were scored as 2+ & 34 (45.3 %) were scored as 3 + .

**Fig. 1:**
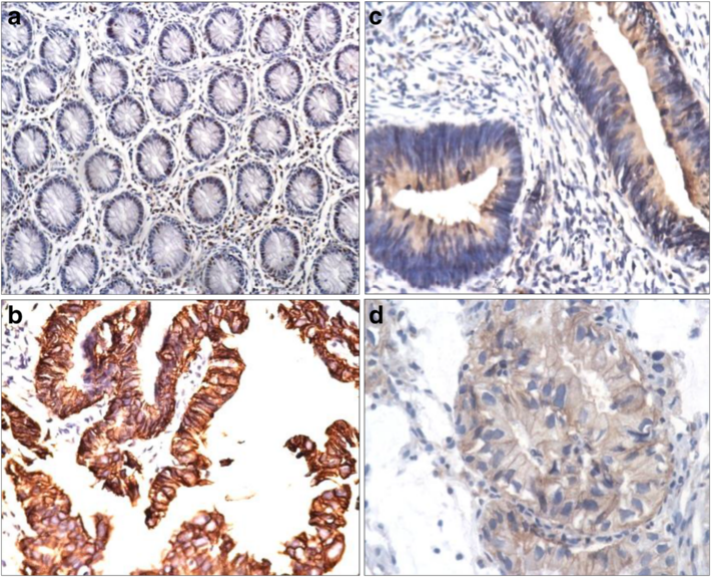
**a** Control (normal colonic mucosa). **b** Membranous expression of Her-2/*neu* in colorectal adenocarcinoma. **c** Cytoplasmic expression of Her-2/*neu* in colorectal adenocarcinoma. **d** Membranous + cytoplasmic expression of Her-2/*neu i*n colorectal adenocarcinoma

A statistically significant difference was seen between pattern of Her-2*/neu* staining & grades of colorectal cancer (*p*-value = 0.038) (Fig. 1). Membranous Her-2/*neu* staining was maximum in high grade while cytoplasmic staining was more frequent in low grade colorectal cancer. Moreover, we observed that the frequency of membranous Her-2/*neu* over expression increased with grades of tumor while cytoplasmic over expression decreased from low to high grade colorectal cancer. A statistically significant association was observed between pattern of Her-2/*neu* staining & type of colorectal cancer depicting membranous Her-2/*neu* staining more common in mucinous while cytoplasmic more frequent in non mucinous type of colorectal cancer (*p*-value = 0.024). No significant difference was noted between pattern of Her-2/*neu* staining with age of the patient & stage of the tumor (Table 1).

**Table 1:** Pattern of Her-2 staining in colorectal cancer

			Pattern of Her-2 staining n(%)				*p*-value
			Negative	Memb	Cyto	M + C	
Gender	F	44	7 (15.9)	4 (9.1)	21 (47.7)	12 (27.3)	0.013*
	M	51	13 (25.5)	16 (31.4)	15 (29.4)	7 (13.7)	
Age	≤60 year	70	15 (21.4)	16 (22.9)	24 (34.3)	15 (21.4)	0.666*
	>60 year	25	5 (20.0)	4 (16.0)	12 (48.0)	4 (16.0)	
Grade	I	14	3 (21.4)	1 (7.1)	7 (50.0)	3 (21.8)	0.038**
	II	59	11 (18.6)	9 (15.3)	25 (42.4)	14 (23.7)	
	III	22	6 (27.3)	10 (45.5)	4 (18.2)	2 (9.1)	
Type	Mucinous	13	5 (38.5)	5 (38.5)	1 (7.7)	2 (15.4)	0.024**
	Nonmucinous	82	15 (18.3)	15 (18.3)	35 (42.7)	17 (20.7)	
pTNM	IIA	9	3 (37.5)	2 (50.0)	1 (80.0)	0	0.116**
	IIB	1	1 (100)	0	0	0	
	IIIA	2	2 (100)	0	0	0	
	IIIB	3	1 (33.3)	1 (33.3)	1 (33.3)	0	
	IIIC	6	1 (16.7)	2 (33.3)	0	3 (50.0)	

*Pearson Chi square, ** Fisher’s exact, level of significance at < 0.05

(Age ≤ 60 = age less than or equal to 60 years)

There was no significant association between intensity of Her-2/*neu* staining with clinicopathological variables (Table 2).

**Table 2:** Intensity of Her-2 staining in colorectal cancer

			Intensity of Her-2 staining *n*(%)				*p*-value
			Negative	Weak	Mod	Strong	
Gender	F	44	7 (15.9)	9 (20.5)	20 (45.5)	8 (18.2)	0.353*
	M	51	13 (25.5)	10 (19,6)	15 (29.4)	13 (25.5)	
Age	≤60 year	70	15 (21.4)	12 (17.1)	25 (35.7)	18 (25.7)	0.438*
	>60 year	25	5 (20.0)	8 (32.0)	8 (32.0)	4 (16.0)	
Grade	I	14	3 (21.4)	4 (28.6)	4 (28.6)	3 (21.4)	0.514*
	II	59	11 (18.6)	9 (15.3)	23 (39.0)	16 (27.1)	
	III	22	6 (27.3)	6 (27.3)	8 (36.4)	2 (9.1)	
Type	Mucinous	13	5 (38.5)	3 (23.1)	4 (30.8)	1 (7.7)	0.294**
	Nonmucinous	82	15 (18.3)	16 (19.5)	31 (37.8)	20 (24.4)	
pTNM	IIA	9	3 (33.3)	3 (33.3)	3 (33.3)	0	0.267**
	IIB	1	1 (100)	0	0	0	
	IIIA	2	2 (100)	0	0	0	
	IIIB	3	1 (33.3)	1 (33.3)	1 (33.3)	0	
	IIIC	6	1 (16.7)	1 (6.7)	1 (16.7)	3 (50.0)	

*Pearson Chi square, ** Fisher’s exact, level of significance at < 0.05

(Age ≤ 60 = age less than or equal to 60 years)

We observed a significant association between percentage of cells stained & tumor type, with score 3+ maximum in non mucinous type of colorectal cancer (*p*-value = 0.006 (Table 3)).

**Table 3:** Percentage of Her-2 staining in colorectal cancer

			Her-2 score *n* (%)				*p*-value
			0	1+	2+	3+	
Gender	F	44	7 (15.9)	10 (22.7)	10 (22.7)	17 (38.6)	0.592*
	M	51	13 (25.5)	13 (25.5)	8 (15.7)	17 (33.3)	
Age	≤60 year	70	15 (21.4)	14 (20.0)	15 (21.4)	26 (37.1)	0.408*
	>60 year	25	5 (20.0)	9 (36.0)	3 (12.0)	8 (32.0)	
Grade	I	14	3 (21.4)	4 (28.6)	1 (7.1)	6 (4.9)	0.627**
	II	59	11 (18.6)	12 (20.3)	13 (22.0)	23 (39.0)	
	III	22	6 (27.3)	7 (31.8)	4 (18.2)	5 (22.7)	
Type	Mucinous	13	5 (38.5)	5 (38.5)	3 (23.1))	0	0.006**
	Nonmucinous	82	15 (18.3)	18 (22.0)	15 (18.3)	34 (41.5)	
pTNM	IIA	9	3 (33.3)	4 (44.4)	2 (22.2)	0	0.028**
	IIB	1	1 (100)	0	0	0	
	IIIA	2	2 (100)	0	0	0	
	IIIB	3	1 (33.3)	0	2 (66.7)	0	
	IIIC	6	1 (16.7)	1 (16.7)	0	4 (66.7)	

*Pearson Chi square, ** Fisher’s exact, level of significance at < 0.05

(Age ≤ 60 = age less than or equal to 60 years)

## Discussion

In this study our focus was to evaluate Her-2*/neu* expression in colorectal adencarcinoma and to correlate it with various clinicopathological variables. Various research groups have investigated the expression patterns of Her-2/*neu* in colorectal carcinomas with a variability ranging from 0 to 84 % [9]. Average membranous expression of Her-2*/neu* in colorectal cancer is about 5 % while cytoplasmic expression ranges between 0-66 % with an average of 30 % [9]. These variable data could be attributed to several factors including use of different antibodies, different sample size, and use of non-uniform scoring system for interpretation of results amongst others [9]. We have highlighted some of the studies done in the past which observed correlation between Her-2/*neu* expression in colorectal cancer & grade and stage of tumor using different antibodies (Table 4). Hence, to avoid the inconsistency in results and for better reporting of Her-2/*neu* expression in colorectal carcinoma a standardized protocol is urgently in need. In breast cancer, Her-2/*neu* scoring follows Hercep test where membranous expression of Her-2/*neu* is evaluated by a cumulative score based on intensity of reactivity, complete or incomplete staining & percentage of cells stained [18]. In gastric cancer, Hofmann Validation scoring is recommended for membranous expression of Her-2/*neu* [19]. Whereas in colorectal cancer, a validated scoring has not been established yet. However, there are few studies which followed the three criterias related to Her-2/*neu* staining pattern, intensity and percentage of cells stained [15, 17, 20].

**Table 4:** Correlation of Her-2+/neu cases of colorectal carcinoma with tumor grade & stage

Author	Antibody used	Samples (n)	Her-2 + ve (%)			Grade	Stage
			M	C	M + C		
Seo et al. [26]	Monoclonal 4B5	365	6	-	-	NA	NA
Gill et al. [15]	Monoclonal RTU-CB11	40	-	57.5	7.5	II/III	III/IV
Li et al. [27]	Hercep test Kit	317	15.4	-	-	NS	III
Anwar et al. [13]	Monoclonal PY1248	100	42	-	-	I/II	NS
Kruszweski et al. [21]	Polyclonal A0485	202	26.7	66.3	-	NA	NA
Kafi et al. [17]	Polyclonal K5204	69	-	27	14	I	NA
Tavangar et al. [28]	Monoclonal	55	-	-	21.8	II/III	III

*M* membranous, *C* cytoplasmic, *M + C* membranous + cytoplasmic expression, *NA* not associated, *NS* not stated in the manuscript

Our study observed majority of colorectal cancers with cytoplasmic expression of Her-2/*neu* (48 %) which corresponds to the studies which followed all the three parameters of Her-2/*neu* evaluation [15, 17, 20, 21]. In our series we found 26.6 % positivity for membranous Her-2/*neu* staining. On contrary to this, two studies from the same region observed higher percentage of membranous expression of Her-2/*neu* in colorectal carcinomas, 74.1 % (*n* = 31) and 42 % (*n* = 100) respectively [13, 22, 23]. The reason for the difference in the rate might be due to different scoring protocols where they took into account only the membranous expression of Her-2*/neu* staining and did not consider the cytoplasmic expression. In the present study, we found a significant association between pattern of Her-2/*neu* staining & grade of colorectal cancer which is in accordance with one of the study done in the same region but to note here is that they compared the significance of Her-2/*neu* expression in different grades amongst different types of gastrointestinal carcinomas (gastric, small intestine and colorectal carcinomas) and did not primarily correlate the Her-2/*neu* expression within different grades of colorectal carcinoma. Similarly, the other study done in our area reported a significant membranous Her-2/*neu* expression in low grade colorectal carcinoma [13]. On contrary to this we noted membranous Her-2/*neu* expression to be significantly associated with high grade colorectal cancer. As mentioned earlier that these disagreements might be due to difference in scoring protocol, where they observed only membranous staining.

A study from West, conducted on 121 colorectal cancer cell lines for Her-2/*neu* expression, observed 63.5 % cases with cytoplasmic expression and a significant association with low grade colorectal carcinoma [24]. Their finding was similar to ours with respect to association with grade of colorectal cancer. While, study from Iran & India found significant association of cytoplasmic Her-2 expression with high grade (100 %). This discrepancy might be due to the fact that they had only two cases classified under high grade cancer [15, 20, 24].

A very few studies attempted to study the intensity & percentage of Her-2/*neu* staining where as similar to our study they also depicted insignificant findings [15, 17, 20]. These studies suggest that in future focus should be made on the pattern of Her-2/*neu* staining in colorectal carcinomas. Membranous Her-2/*neu* expression in colorectal cancer could be a future extracellular therapeutic target by Trastuzumab. An intracellular kinase inhibitor, Lapatinib has been recently approved for Trastuzumab resistant breast cancer patients [25]. Assuming the role of cytoplasmic Her-2*/neu* expression in pathogenesis of colorectal carcinoma, Lapatinib could be advancement in the treatment of colorectal cancer patients.

One of the limitation of our study was small sample size of colectomy cases (*n* = 21) to investigate the association between pTNM & Her-2/*neu* expression. Although, a significant association is seen between percentage of cells stained with Her-2/*neu* & stage of tumor, this might have low statistical power. Nevertheless, our study is an extensive effort done in South Asia region which observed all the three parameters of Her-2*/neu* expression in colorectal carcinoma.

## Conclusion

Our study concludes a high expression of Her-2/*neu* in colorectal cancer in our population. A significant strong association of cytoplasmic Her-2/*neu* expression with low grades whereas membranous Her-2/*neu* expression with high grade colorectal cancer. These findings may help in conducting clinical trials in future to explore its therapeutic significance as well.

## Abbreviations

DDRRL: Dow Diagnostic Research and Reference Laboratory
DUHS: Dow University of Health Sciences
H&E: Hematoxylin & eosin
Her-2: Human Epidermal Growth Factor Receptor
NILGID: National Institute of Liver & Gastrointestinal Diseases
WHO: World Health Organization
